# Structure and regulation of violaxanthin de-epoxidase, a key enzyme in the photoprotection of photosynthesis

**DOI:** 10.1093/plphys/kiag224

**Published:** 2026-04-16

**Authors:** Anna Santin, Eleonora Mezzadrelli, Giorgio Perin, Tomas Morosinotto

**Affiliations:** Department of Biology, University of Padova, Padova 35131, Italy; Department of Biology, University of Padova, Padova 35131, Italy; Department of Biology, University of Padova, Padova 35131, Italy; Department of Biology, University of Padova, Padova 35131, Italy

## Abstract

The xanthophyll cycle is a set of light dependent reactions that regulate the conversion of violaxanthin into zeaxanthin, representing a key mechanism in the regulation of photosynthesis and the response to excess light in eukaryotes. Optimization of zeaxanthin accumulation has also been identified as a promising target for increasing photosynthetic productivity in crops, balancing the trade-off between photoprotection and photosynthetic efficiency. The activation of the xanthophyll cycle depends on violaxanthin de-epoxidase (VDE), the enzyme that catalyzes the conversion of violaxanthin into zeaxanthin, activated under conditions of light excess. Photosynthetic eukaryote genomes contain other genes with similarity to VDE, called VDL and VDR, that show conserved structural properties but have different biological roles. The available knowledge on VDE structure and catalytic mechanism is reviewed here, including the identification of key amino acids involved in the catalytic mechanisms and conformational changes during protein activation. VDE activity is also shown to be modulated by multiple mechanisms, such as transcriptional control, redox sensitivity, and pH-dependence. All these regulatory mechanisms have an essential role in modulating VDE protein activity in vivo, and their impact should be considered in efforts to optimize photosynthetic productivity.

## Teaser

Violaxanthin de-epoxidase controls the conversion of light-sensitive pigments, balancing energy capture and protection from light stress in plants and algae.

## Advances

- VDE has a major role in photosynthesis regulation in eukaryotes.
- Genetic engineering efforts demonstrated that VDE is a potential target to optimize light-use efficiency and photosynthetic productivity in plants and microalgae.
- Structural and biochemical studies clarified the VDE reaction mechanism and enabled the identification of key amino acids involved in catalytic mechanisms and conformational change during protein activation.
- The genomes of photosynthetic eukaryotes present other genes with similarity to VDE, called VDL and VDR, that show conserved structural properties but distinct regulation and biological role.
- VDE activity is controlled at multiple levels, such as transcription, post-translation and substrate availability, linking environmental cues to photosynthesis modulation.

## Outstanding questions

- What are the structural properties and functional role of VDE N- and C-terminal domains?
- Is there a significant functional diversity of VDE from different phylogenetic group?
- Does the modulation of VDE oxidation state play a role in regulating the enzymatic activity in vivo?
- How does VDE interact with the thylakoid membranes?
- How much can targeted manipulation of VDE regulation impact biomass productivity in crops and microalgae?

## The xanthophyll cycle is a key mechanism for the regulation of photosynthesis in eukaryotes

Photosynthetic organisms exploit solar energy to fuel their metabolism. In a highly dynamic natural environment, however, light availability is not constant, generating a significant physiological challenge (Takahashi and Badger 2011): while the maximization of light harvesting is essential to sustain the photochemical reactions of photosynthesis, excess light can saturate photochemistry and damage the photosynthetic apparatus (Pinnola and Bassi 2018). Photodamage can occur because the accumulation of singlet chlorophyll excited states (^1^Chl*) can generate triplet chlorophyll (^3^Chl*), which reacts with molecular oxygen (O_2_), to form reactive oxygen species (ROS) such as singlet oxygen (^1^O_2_*) (Tjus et al. 2001; Dmitrieva et al. 2020). Moreover, the overreduction of the photosynthetic electron transport chain can reduce O_2_ to superoxide (O_2_^−^), particularly when electron acceptors are limiting (Bassi and Dall’Osto 2021). Both processes lead to oxidative damage of pigments, proteins and lipids, thus reducing photosynthetic efficiency (Takahashi and Badger 2011), a condition known as photoinhibition.

All photosynthetic organisms have evolved photoprotective mechanisms that mitigate these detrimental effects at both the molecular and physiological levels. Antioxidant systems play a pivotal role in directly scavenging ROS and maintaining redox homeostasis under high light exposure (Foyer and Shigeoka 2011; Rao et al. 2025). State transitions instead facilitate the redistribution of excitation energy between Photosystem II (PSII) and I (PSI), balancing the photosynthetic electron transport chain and preventing the overreduction of the photosynthetic apparatus (Minagawa 2011; Nawrocki et al. 2016). Alternative electron flows, such as cyclic and pseudo-cyclic electron transport, also serve as safety valves, lowering the chances of electron transport overreduction and minimizing photodamage especially at the level of PSI (Eberhard et al. 2008; Shikanai 2014; Saroussi et al. 2019). A central component of the photoprotection network is the so-called non-photochemical quenching (NPQ), which allows photosynthetic organisms to dissipate excess excitation as harmless thermal energy in the light-harvesting complexes of PSII (Müller et al. 2001; Li et al. 2009). NPQ acts by quenching excess ^1^Chl* excited states, thus lowering the chance of generating harmful ^3^Chl* (Carbonera et al. 2012). NPQ depends on the presence of species-specific protein activators, PsbS in plants and LHCSR/LHCX in eukaryotic microalgae, which are activated by the decrease in the pH of the thylakoid lumen to induce the thermal dissipation of excess energy (Demmig-Adams et al. 2020; Bassi and Dall’Osto 2021). NPQ is a very fast mechanism for the modulation of photosynthesis that develops within seconds upon an increase in light intensity, in response to a trans-thylakoid pH gradient (Niyogi 2000), and most of it relaxes within 1 to 2 minutes upon return to low light (Demmig-Adams et al. 1996).

**Figure 1:**
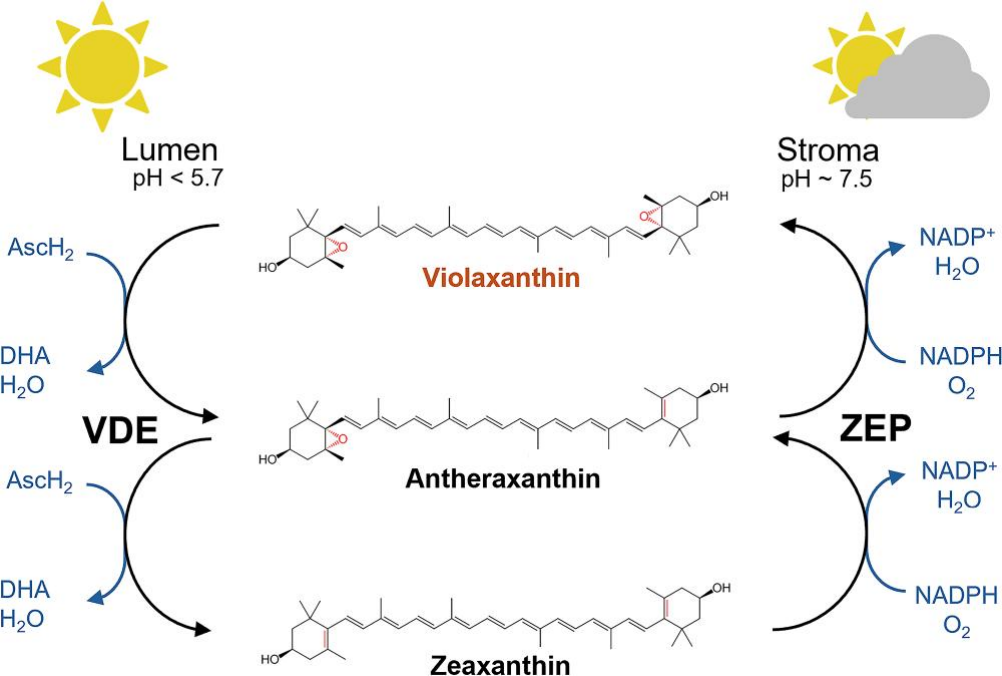
Reactions and enzymes involved in the xanthophyll cycle. Violaxanthin de-epoxidase (VDE) de-epoxidizes violaxanthin to antheraxanthin and then to zeaxanthin, also converting two ascorbic acid molecules into dehydroascorbate. De-epoxidation is induced under high illumination by the decrease of the thylakoidal lumen pH. Zeaxanthin Epoxidase (ZEP) instead catalyzes the inverse reaction, re-generating violaxanthin using O_2_ and NADPH (Büch et al. 1995)/reduced ferredoxin (Bouvier et al. 1996) as electron donors and FAD as cofactor. ZEP activity is believed to be constitutive with its impact being stronger when VDE is inhibited, thus in low light or darkness. The panel represents structures of xanthophylls, co-factors and location of enzyme activities. AscH_2_: ascorbic acid, DHA: dehydroascorbate, Fd: ferredoxin.

Another major photoprotection mechanism is the xanthophyll cycle. Found in all plants and several eukaryotic algae, this mechanism consists of the reversible conversion of violaxanthin (Vx) into zeaxanthin (Zx) operated by two enzymes: violaxanthin de-epoxidase (VDE) and zeaxanthin epoxidase (ZEP) (Fig. 1). Zx synthesis through VDE is induced under excess light, and its accumulation enables maximal NPQ activation in plants (Pinnola et al. 2013). In several organisms where NPQ is LHCSR/LHCX-dependent, such as microalgae and non-vascular plants, NPQ is even more strongly dependent on Zx accumulation (Bailleul et al. 2010; Buck et al. 2019; Michelberger et al. 2025a) even though there are also species where the impact is smaller, like *Chlamydomonas* (Quaas et al. 2015). Zx accumulation was also shown to have a role in the oxidative stress response (Latowski et al. 2013), acting as a scavenger of ^3^Chl* and ROS, with an impact beyond its activity in NPQ (Havaux et al. 2007; Latowski et al. 2013).

Upon exposure to non-saturating light, the epoxidized Vx binds to Light-Harvesting Complexes (LHCs), where it supports light harvesting (Morosinotto et al. 2002). In this condition, VDE is found as a soluble and inactive monomeric enzyme in the thylakoid lumen. Upon excess light exposure, and the subsequent increased acidification of the thylakoid lumen (Gilmore et al. 1998; Arnoux et al. 2009; Blommaert et al. 2021), VDE dimerizes and interacts with thylakoid membranes where it binds Vx (Yamamoto and Higashi 1978; Hager and Holocher 1994; Arnoux et al. 2009), which was previously released from antenna proteins. Here Vx is de-epoxidized, forming the semi-epoxidized intermediate antheraxanthin (Ax) and the fully de-epoxidized Zx (Demmig-Adams et al. 2020; Fernández-Marín et al. 2021) (Fig. 1). The intermediate Ax, which is transiently accumulated, has also been proposed to contribute to photoprotection (Goss et al. 1998; Short et al. 2023).

Vx de-epoxidation involves the reduction of an epoxide group at both ends of the molecule, during which the oxygen atom is released, and a double bond is formed. For each epoxide group the reducing power is provided by a molecule of ascorbic acid, which acts as an electron donor, and a molecule of water is released (Jahns et al. 2009) (Fig. 1). Vx de-epoxidation was observed experimentally in spinach thylakoid membranes upon artificial acidification, which induced the activation of VDE, leading to the consequent activation of NPQ (Dlouhý et al. 2020). In fact, once synthesized, Zx can rebind to the LHCs, where it triggers their reorganization into a quenched state (Johnson et al. 2011) that facilitates the dissipation of excess excitation energy as heat (Azadi-Chegeni et al. 2022). When light intensity decreases, the opposite reaction occurs and Zx is re-epoxidized to Ax and then to Vx by the ZEP enzyme (Büch et al. 1995; Bouvier et al. 1996), ensuring Vx turnover and maintaining the size of the Vx pool (Eskling and Åkerlund 1998; Jahns et al. 2009; Pinnola and Bassi 2018; Bassi and Dall’Osto 2021). ZEP activity is also modulated by enzyme expression levels and redox state, impacting the Vx/Zx balance (Jahns and Bethmann 2025).

## Genetic control of VDE activity for the optimization of photosynthesis yield

Because of its pivotal role in regulating photosynthesis, VDE has been identified as a target for the genetic improvement of light-use efficiency in photosynthetic eukaryotes. Tuning VDE activity, in combination with other genes that directly (ie ZEP) and indirectly (ie light-harvesting proteins) regulate the xanthophyll cycle (Fig. 1), has recently been proposed as a strategy to optimize photosynthetic efficiency. The upregulation of VDE, PsbS, and ZEP significantly accelerated xanthophyll interconversion, leading to faster induction and relaxation of NPQ and increased productivity in the field in both tobacco and soybean (Kromdijk et al. 2016; De Souza et al. 2022). However, similar approaches did not generate the same results in other species, eg *Arabidopsis* (Garcia-Molina and Leister 2020) and potato (Lehretz et al. 2022), indicating that species-specific physiological or morphological features are highly influential.

The genetic manipulation of the xanthophyll cycle has been shown to potentially increase biomass productivity also in marine microalgae of the genus *Nannochloropsis* cultivated in photobioreactors (Perin et al. 2023). Also in the case of microalgae, results between species are not always equivalent (Manfellotto et al. 2020), suggesting again that the impact of such an approach is affected by other species-specific variables also in photosynthetic microorganisms. In the seawater alga *Nannochloropsis*, the overexpression of VDE and ZEP resulted in markedly accelerated xanthophyll cycle activity and NPQ induction and relaxation across a range of light conditions (Michelberger et al. 2025a). The impact on biomass productivity, however, depended on the growth conditions. In limiting light, accelerated Zx biosynthesis came at the expense of photosynthetic efficiency, while too fast xanthophyll cycle relaxation caused increased photosensitivity at higher illumination. These results highlight the existence of a physiological trade-off between photosynthetic efficiency and photoprotection, where the optimal balance can vary depending on the light conditions (Jahns and Bethmann 2025; Michelberger et al. 2025a; Michelberger et al. 2025b).

Overall, these findings show that it is indeed possible to increase biomass productivity by modifying xanthophyll cycle dynamics and emphasize that VDE is a valuable target for optimizing photosynthesis to the light dynamics found in crop fields or microalgae cultivated in photobioreactors. The results are however highly influenced by factors such as the variability of the impact of Zx on energy dissipation (Quaas et al. 2015), species-specific features such as canopy structure and external factors such as environmental conditions. To maximize the photosynthetic potential, it is thus necessary to consider the complexity of photosynthesis regulation and its interaction with environmental dynamics. Therefore, all layers of regulation of VDE activity must be fully understood and considered in biotechnological improvement efforts.

## Diversity of VDE across photosynthetic eukaryotes

The light regulated xanthophyll cycle is widely distributed among photosynthetic eukaryotes that rely on a transmembrane LHC as antennae, while it is absent in all prokaryotes, Cryptophytes, Glaucophytes, and Rhodophytes, which instead rely on soluble phycobilisomes (Jahns et al. 2009; Goss and Lepetit 2015). As discussed above, in land plants de-epoxidation of Vx to Zx is activated upon high light, through the so-called Vx-cycle (Yamamoto et al. 1962; Niyogi 2000). At least two other types of xanthophyll cycles have been identified in various organisms. An alternative xanthophyll cycle, involving lutein (Lx) and Lx-epoxide, was found in some shade-adapted or parasitic plants, such as *Cuscuta reflexa*, *Amyema miquelii*, and *Quercus* sp. (Bungard et al. 1999; García-Plazaola et al. 2007; Jahns et al. 2009), possibly providing an advantage in these peculiar ecological niches (Matsubara et al. 2007). Some eukaryotic algae, including dinoflagellates and diatoms, present instead the Diadinoxanthin (Ddx)-cycle (Stransky and Hager 1970; Coesel et al. 2008), involving the conversion of Ddx into Diatoxanthin (Dtx) (Lavaud et al. 2002). No form of the xanthophyll cycle has been observed to date in Cryptophytes, Glaucophytes, and Rhodophytes (Jahns et al. 2009; Jinkerson et al. 2024). This is likely because as PSII antennas they rely on phycobilisomes that do not bind xanthophylls (Gantt et al. 2003), suggesting that alternative strategies for photoprotection have evolved in these lineages (Esteban et al. 2009).

**Figure 2:**
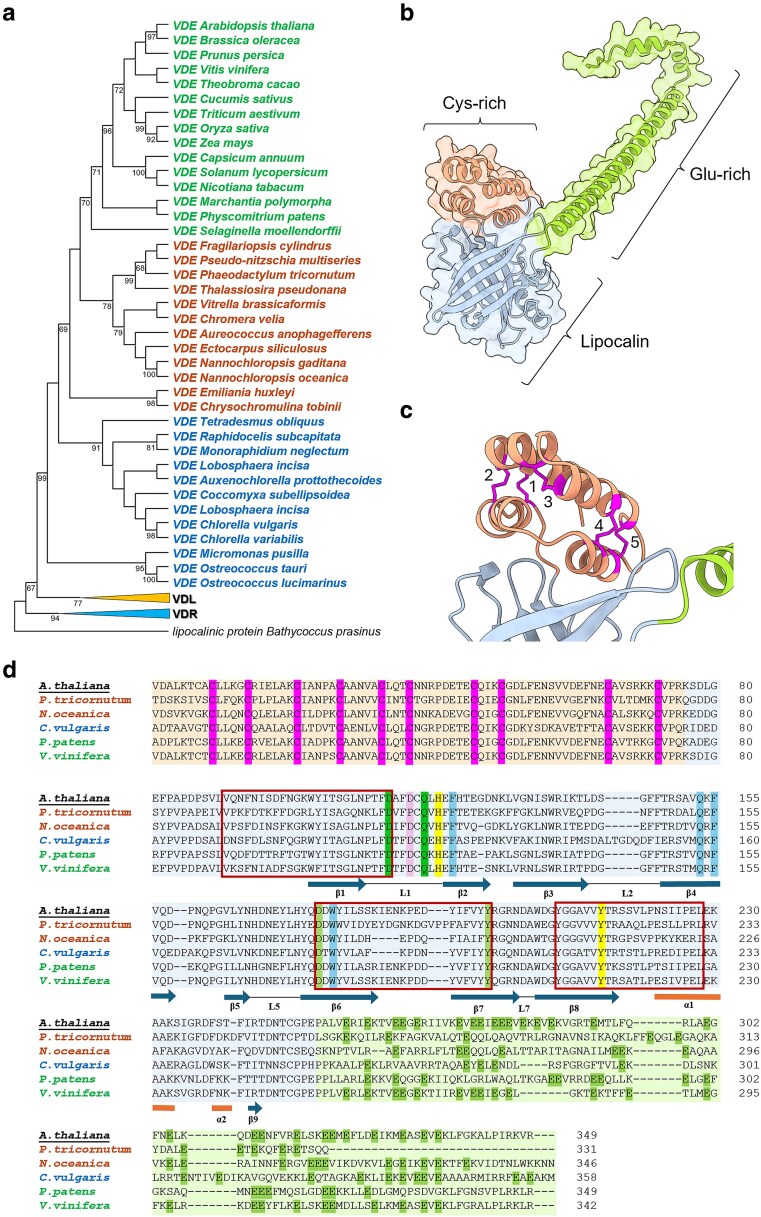
VDE evolutionary and structural conservation. **(a)** Phylogenetic tree of VDE proteins from different lineages: land plants shown in green, SAR (Stramenopiles, Alveolates and Rhizarians, secondary endosymbionts) in orange and green algae in blue. Maximum-Likelihood phylogenetic tree was obtained by multiple alignment of protein sequences obtained from Coesel et al. (2008); Girolomoni et al. (2020) and Dautermann et al. (2020). Bootstrap values (/100) are reported on significant nodes. *Bathycoccus prasimus* lipocalin domain was used as outgroup. VDL: VDE-like proteins, VDR: VDE-related proteins. **(b)**  *Arabidopsis thaliana* VDE structure (predicted by AlphaFold, AF-Q39249-F1, based on *A. thaliana* VDE structure reported from UniProt, Q39249) with the three different domains highlighted with different colors: N-terminal Cys-rich domain in orange, the central lipocalin domain in light blue and the C-terminal domain in green. The structural organization of the latter domain is predicted with low confidence. **(c)** Detail of the Cys-rich domain showing the ten conserved Cys residues highlighted in pink: predicted disulfide bonds between Cys based on the relative distance are shown as sticks and labeled according to numbering as (1) C9-C72, (2) C14-C65, (3) C21-C27, (4) C33-C50, (5) C37-C46. **(d)** Sequence alignment of VDE enzymes from different representative photosynthetic eukaryotes. Boxes indicate different domains: the N-terminal Cys-rich domain in orange, the central lipocalin domain in light blue and the C-terminal domain in green. Conserved residues are color-coded according to the domain organization: pink for the 10 cysteine residues on the N-terminal domain. In the lipocalin domain: light green for the key residues involved in primary VDE catalytic activity associated with Violaxanthin (Vx), blue for key residues stabilizing Vx binding, yellow for residues involved in pH sensing and VDE conformational change, pink for residues involved in priming acidic-to-neutral pH transition. Dark green for the Glu-residues in the C-terminal domain. Red squares indicate conserved lipocalin motifs. Secondary structure features are shown and labeled below the alignment (orange cylinders indicate α-helices and blue arrows β-sheets, often connected by loops).

Despite this diversity in activity, almost all xanthophyll cycles identified so far rely on homologous VDE enzymes that are identifiable in their genomes (Fig. 2a). VDE from land plants and mosses cluster into a clade clearly distinct from those found in other taxa. A separate clade includes all VDEs from organisms whose plastids originated by secondary (or tertiary) endosymbiosis, including Ochrophytes, such as diatoms and Eustigmatophyceae (eg *Nannochloropsis* spp.), Haptophytes like *Emiliania huxleyi* (now renamed *Gephyrocapsa huxleyi*) and photosynthetic Alveolates such as dinoflagellates and *Chromera velia* (Fig. 2a). All these organisms possess a VDE gene even if the plastid originated from an ancestral Rhodophyte (Morozov and Galachyants 2019; Strassert et al. 2021) that lacked both VDE and any other form of the xanthophyll cycle (Esteban et al. 2009; Jahns et al. 2009), raising an interesting evolutionary question about its origin. One possibility is that Rhodophytes lost VDE during evolution, but this hypothesis seems unlikely since the common ancestor most likely did not have Vx, the enzyme substrate nor LHC, the proteins binding it. It is more likely that the VDE enzymes present in secondary endosymbionts are the result of horizontal gene transfer. The latter hypothesis is also consistent with the observation that VDE proteins from secondary endosymbionts do not cluster separately from those in plants and green algae. This suggests that the former may have acquired the gene after the divergence between Chlorophytes (ie green algae) and Streptophytes (ie land plants), thus supporting the hypothesis of horizontal transfer (Fig. 2a). Resolving the precise evolutionary origin of this enzymatic function remains an intriguing open question in understanding the evolution of xanthophyll cycle enzymes.

VDE homologs can be found in most green algae, and the sequences form a distinct clade from plants even though most green algae possess a functional Vx-cycle (Girolomoni et al. 2020; Tóth 2023). It is however worth remarking that in some Volvocales species, such as *Chlamydomonas reinhardtii* and *Volvox carteri*, the canonical VDE is replaced by another protein called Chlorophyta VDE (CVDE), which has no homology with VDE, is localized in the stroma and whose activity is independent of lumenal acidification (Li et al. 2016). Phylogenetic analyses suggest that CVDE evolved from an ancient de-epoxidase, showing similarity to enzymes involved in bacterial carotenoid biosynthesis, such as lycopene cyclase (Maresca et al. 2007; Li et al. 2016). This peculiar case further emphasizes the biological relevance of de-epoxidation in photosynthetic eukaryotes, since the eventual evolutionary loss of VDE required the selection of an alternative protein to replace its activity.

Finally, it is worth mentioning that the VDE forms involved in the Vx cycle are found in each of the three phylogenetic groups shown in Fig. 2a, including plants, secondary endosymbionts (eg *Nannochloropsis* spp.; Michelberger et al. 2025a) and green algae (eg *Chlorella vulgaris*; Girolomoni et al. 2020). The tree in Fig. 2a also includes proteins from diatoms that are involved in the Diadinoxanthin (Ddx) cycle. This shows that the diversity in xanthophyll cycles is not strictly associated with the diversity in VDE properties and that the same protein participates in different xanthophyll cycles, due to the enzyme's promiscuous reactivity with several different carotenoids (Yamamoto and Higashi 1978).

## VDE structural organization

The VDE is a nuclear encoded protein organized into three domains: an N-terminal cysteine (Cys)-rich domain, a central lipocalin domain and a C-terminal glutamic acid (Glu)-rich domain (Fig. 2b) (Hieber et al. 2002). The Cys-rich domain is about 80 residues long and contains 10 Cys residues highly conserved among different species (Hieber et al. 2002; Hallin et al. 2016a) (Fig. 2c, d). Even if it is likely not directly involved in the catalytic activity, this domain is essential, and its partial deletion leads to protein inactivation (Hieber et al. 2002). Even the mutation of individual cysteines caused drastic negative effects on protein activity (Simionato et al. 2015). These results, together with the observation that reducing agents, such as DTT, fully inhibit VDE (Hieber et al. 2002), strongly suggest that these cysteines are involved in several disulfide bridges essential for the protein activity. It is worth underlining that, while it is clear that VDE is active when oxidized, it has never been shown that its activity is regulated in vivo by the modulation of its redox state, while this is well established for other chloroplast proteins localized in the stroma such as Calvin Benson cycle enzymes (Buchanan 1991).

The C-terminal domain is about 100 amino acids long and contains a high number of Glu residues. It shows low similarity with other known proteins and its structure is predicted with low confidence (Fig. 2b). While the richness of acidic residues is shared by all VDE protein sequences, their positions are not equally conserved (Fig. 2d). This domain was proposed to be involved in the interaction of the enzyme with the thylakoid membrane at low pH, due to the partial protonation of Glu residues (Hieber et al. 2002), even though this has never been experimentally demonstrated. Circular dichroism also suggested that the C-terminus could be involved in the assembly of VDE oligomers by forming coiled-coil structures when the protein is in its active form (Hallin et al. 2016b).

The central domain shows homology to lipocalins (Fig. 2b), a large family of small proteins found in many different organisms. Most lipocalins are involved in the passive transport of hydrophobic molecules, but there are a few cases in plants (Hofmann et al. 2006), bacteria (Bishop 2000), and animals (Clayton et al. 2019), where they have enzymatic activity, such as both enzymes involved in the xanthophyll cycle, VDE and ZEP (Hieber et al. 2000). Members of the lipocalin family share low sequence identity, except for three short motifs (Hieber et al. 2000), but they present a conserved three-dimensional structure. The typical lipocalin, in fact, shows a barrel structure formed by eight antiparallel β-sheets that enclose an internal binding site for the substrate or ligand (Grzyb et al. 2006).

**Figure 3:**
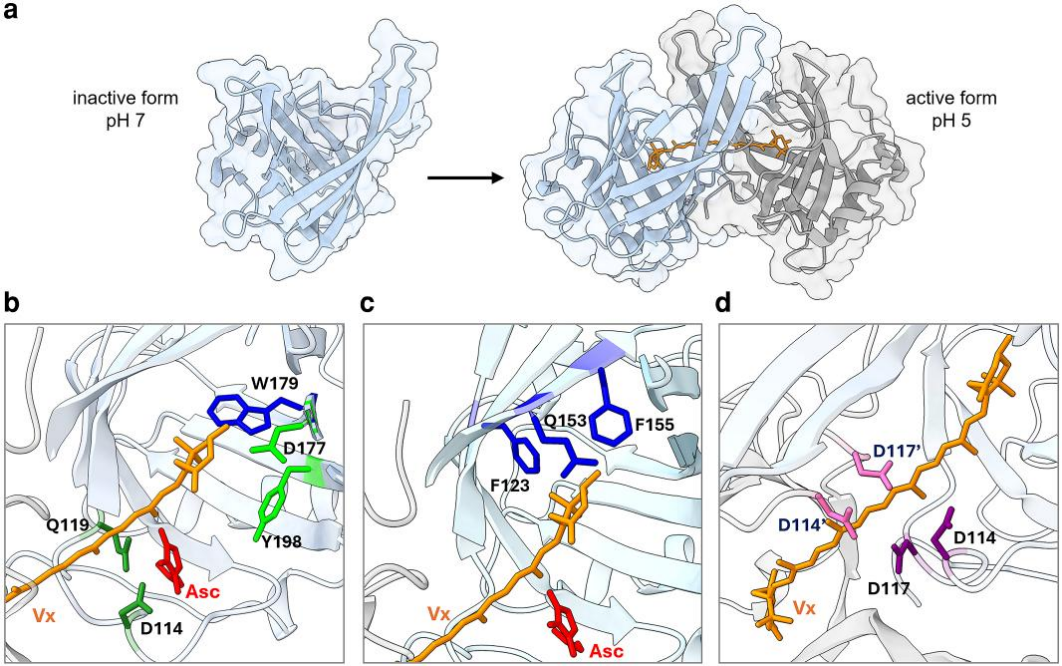
Structural properties of the VDE lipocalin domain and key residues for its activity. **(a)** Scheme of VDE activation through dimerization. At neutral pH VDE lipocalin domain is monomeric (shown light blue), while it shows dimeric form at acidic pH (shown in light gray and blue). Structure in active form was solved at pH 5, but lumenal pH in vivo is not expected to go below 5.5 (Tian et al. 2019). Violaxanthin (Vx), in orange, can accommodate in the cavity generated within the dimer. **(b)** Residues involved in the catalytic activity are reported as sticks: Vx interaction and catalytic activity (light green), substrate binding stabilization (blue) and ascorbic acid (Asc) interaction (dark green). **(c)** Other residues putatively involved in the stabilization of substrate interaction are highlighted in blue. **(d)** Residues involved in conformational changes, leading to enzyme de-activation. Vx is reported in orange while Asc in red.

The structure of the VDE central lipocalin domain from *Arabidopsis thaliana* has been experimentally resolved by X-ray crystallography (Arnoux et al. 2009) and it shows the typical lipocalin β-barrel structure with three conserved motifs, which include the residues 91–114, 177–198 and 208–228 (Fig. 2b, d) (Coesel et al. 2008). Structural data and results from site directed mutagenesis suggest that the VDE lipocalin domain undergoes a conformational change upon pH reduction that allows its dimerization (Arnoux et al. 2009; Fufezan et al. 2012) (Fig. 3a). The analysis of the two conformational states shows that the lipocalin barrel at pH 7 is in a closed state, where D114 is hydrogen bonded to Y198, thereby partially blocking access to the internal binding cavity (Arnoux et al. 2009). After the conformational change, there is a rearrangement of residue interactions with D114 now involved in a salt bridge with R138 and hydrogen bonded to D114 of the adjoining monomer within the dimer, resulting in the opening of a hydrophobic cavity large enough to accommodate one Vx molecule (Arnoux et al. 2009; Saga et al. 2010; Biswal et al. 2023) (Fig. 3a).

Site-directed mutagenesis enabled the identification of two residues within the lipocalin cavity, D177 and Y198, involved in catalytic activity and whose mutation leads to complete protein inactivation (Saga et al. 2010) (Fig. 3b). D177 was calculated to have a pK_a_ of 5.8, and is thus expected to be mostly protonated at pH below 6 when the protein is active, allowing it to act as a proton donor to the Vx epoxy group. This hypothesis matches the Vx binding model, which locates D177 within 3 Å of the epoxy group (Saga et al. 2010). Substrate docking experiments instead suggested that Y198 is involved in the formation of a hydrogen bond with the second substrate, ascorbic acid, enabling efficient electron donation to Vx (Saga et al. 2010) (Fig. 3b).

A tryptophan residue within the cavity, W179, was proposed to strongly contribute to Vx binding through a hydrophobic interaction (Fig. 3b), similarly to other aromatic residues interacting with carotenoid molecules identified in many other carotenoid-binding proteins, such as light-harvesting proteins (Liu et al. 2004; García-Martín et al. 2008; Saga et al. 2010). Consistent with this hypothesis, its mutation caused a strong loss in activity but not a complete inactivation. Docking simulations also predicted the residues F123, F155, and Q153 to be close to the Vx and possibly involved in stabilizing the substrate binding (Fig. 3c). Their mutations led to a partial VDE inactivation, but significant amounts of Zx are still produced, suggesting a non-essential role (Saga et al. 2010).

The second substrate of VDE is ascorbic acid, which provides reducing equivalents for the reaction. Docking simulations and site-directed mutagenesis suggested that ascorbic acid interacts with T112, D114, and Q119 in *A. thaliana* VDE. In particular, VDE activity showed a significant reduction when D114 and Q119 were mutated, supporting their importance for protein activity (Saga et al. 2010) (Fig. 3b). All these residues, putatively involved in carotenoid or ascorbic acid binding, are strongly conserved in all VDE sequences (Fig. 2d), with the exception of T112, where the interaction with the substrate involves the protein backbone and not the side chain (Saga et al. 2010) and can thus be maintained even if mutated to leucine, as found in some protein sequences.

Several other residues have been identified as relevant for protein activity, even if not directly involved in the catalytic cycle or substrate binding. H121 (Figs 2d and 3c) is ideally positioned to act as a trigger of the VDE conformational change: at pH 7, its aromatic ring is uncharged and hydrogen-bonded to the OH group of Y214, while at lower pH, H121 becomes protonated and can no longer form a hydrogen bond (Arnoux et al. 2009). This small reorientation of the side chain pushes the H121 aromatic ring into steric interference with the adjacent L135, positioned on a β-strand, which consequently causes the opening of the barrel side. This, in turn, promotes the exposure of the residues involved in VDE dimerization to the solvent, leading to the conformational change (Arnoux et al. 2009; Saga et al. 2010). Four other residues were also proposed to be involved in pH activation through calculation of p*K*_a_ values, as they are expected to be protonated only when the pH drops below 6: D98, D117, H168, and D206 (Fufezan et al. 2012). When these residues were mutated, the protein showed a decrease in activity even if not a complete deactivation. Also, these residues are not always conserved across different organisms (Fig. 2d) and this diversity has been proposed to be associated with possible differences in pH sensitivity of VDE isoforms from various species. In some diatom species, in fact, VDE has been suggested to have a higher pH optimum enabling significant de-epoxidation activity at pH 7.2 (Jakob et al. 2001; Grouneva et al. 2006; Blommaert et al. 2021), which could be associated with different regulation of the xanthophyll cycle in vivo with respect to plants (Bailleul et al. 2015; Blommaert et al. 2021). It is, however, not possible to draw solid conclusions about evolutionary trends as the diversity of VDE biochemical properties remains underexplored.

A cluster between D114, D117 and the same residues from the nearby monomer (D114′ and D117′, Figs 2d and 3d) was identified as a candidate for priming the acidic-to-neutral pH transition during enzyme deactivation (Arnoux et al. 2009) (Fig. 3d). In fact, such spatial proximity between these aspartate residues is only possible if they are protonated and thus at a low pH. When the pH increases, their deprotonation would destabilize the cluster, promoting the monomerization of the VDE enzyme and its inactivation (Arnoux et al. 2009; Saga et al. 2010) (Fig. 3d).

## VDE-like and VDE-related proteins

**Figure 4:**
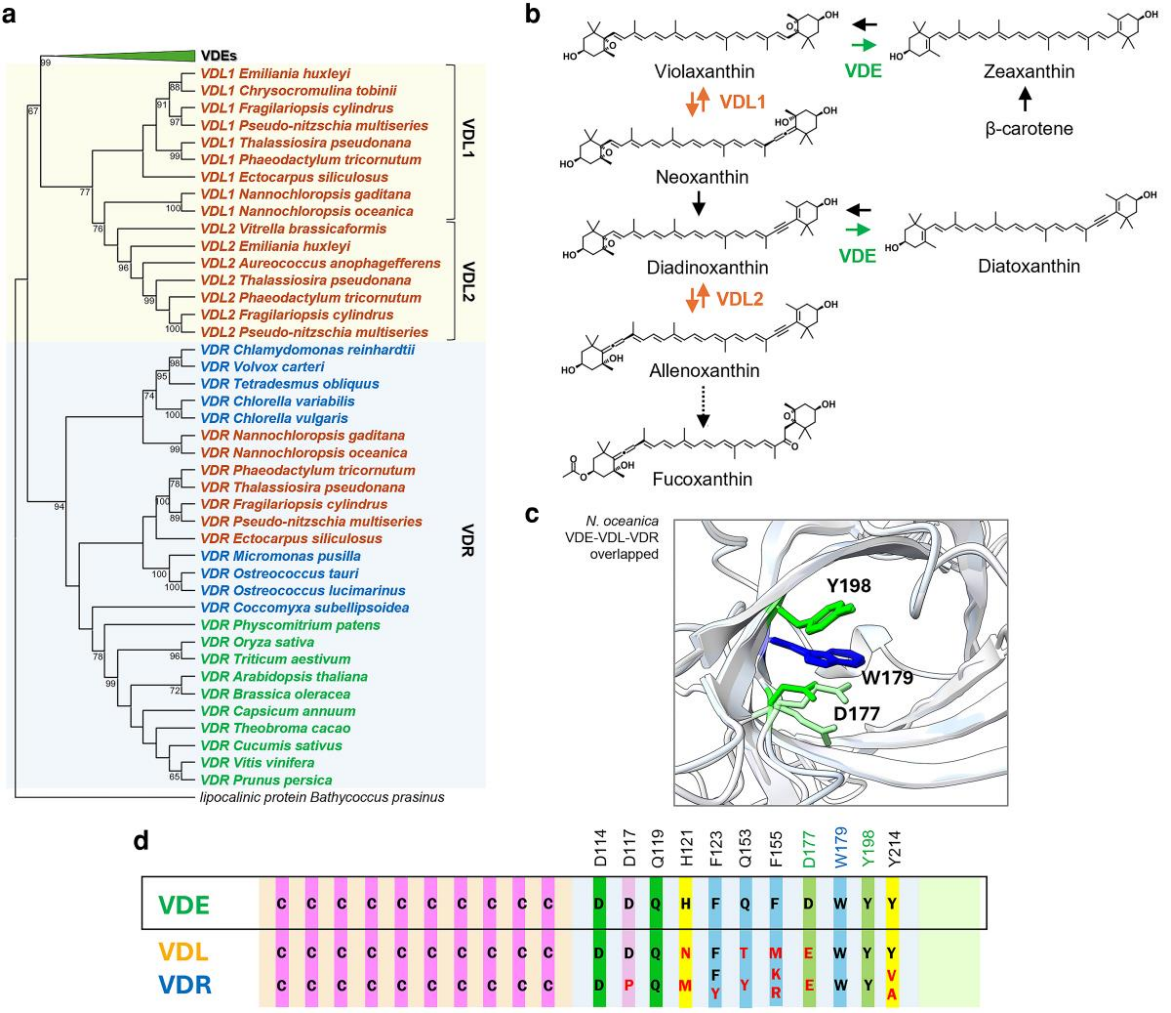
Evolution, function and structure of VDL and VDR proteins. **(a)** Phylogenetic tree showing VDLs in the yellow box and VDR proteins in the light blue box. VDLs divided into two sub-groups: VDL1 and VDL2. The Maximum-Likelihood phylogenetic tree was obtained by multiple alignment of protein sequences obtained from Coesel et al. (2008) and Dautermann et al. (2020). Bootstrap values (/100) are reported on significant nodes. *Bathycoccus prasimus* lipocalinic domain was used as outgroup. **(b)** Schematic carotenoid biosynthesis pathway with experimentally validated reactions catalyzed by VDE and VDLs, with a focus on diatoms that possess both the two VDLs and the VDR. **(c)** Overlap of *N. oceanica* AlphaFold modeled structures of VDE, VDL and VDR, predicted through AlphaFold (model IDs: AF-A0A1B1Q5V0-F1 for VDE, AF-W7TG78-F1 for VDL and AF-W7THW9-F1 for VDR). Key residues involved in the catalytic activity of VDE (green) and its stabilization (blue) are highlighted, including corresponding substitutions with transparent color: W179 and Y198 are conserved while D177 is substituted by a glutamate. **(d)** Conservation of *A. thaliana* VDE key residues in VDE, VDL and VDR, with conserved residues in black while variables in red.

Genome sequence analyses showed that photosynthetic organisms exhibit other proteins similar to VDE, called respectively VDE-related proteins (VDR) and VDE-like proteins (VDL) (Coesel et al. 2008; Girolomoni et al. 2020), which are likely the result of gene duplication events (Wei et al. 2024) (Fig. 4a).

VDRs have been identified across a broad range of taxa, including land plants, mosses, green algae and diatoms (Fig. 4a). This widespread distribution suggests the early emergence and conservation of VDRs within photosynthetic organisms. Even though VDR targeting predictions do not agree for all species, VDRs have been experimentally shown to localize in the chloroplasts in *Cucumis sativus* (Wei et al. 2024). The biological role and eventual substrates of VDR are currently not known, even though *Arabidopsis thaliana* VDR knock-out mutants and *C. sativus* transiently silenced plants suggest a potential involvement in photoprotection under high light (Wei et al. 2024).

VDL sequences are closer to VDE than VDR but are found only within the Chromalveolates, particularly among Stramenopiles (eg *Nannochloropsis* spp. and diatoms), Haptophytes (eg *Emiliania huxleyi*) and Alveolates (Fig. 4a). No VDL forms have been identified in Viridiplantae (Fig. 4a), suggesting that VDLs may have originated after the divergence of the Chromalveolate lineages or were secondarily lost in plants. Notably, diatoms and haptophytes were observed to have two *vdl* genes each (Dautermann et al. 2020; Bai et al. 2022; Giossi et al. 2025). VDL1 has been functionally characterized in the diatom *Phaeodactylum tricornutum* (Li et al. 2024) and in *Nannochloropsis oceanica* (Dautermann et al. 2020), where VDL1 was shown to play a central role in carotenoid biosynthesis, catalysing the tautomerization of Vx to neoxanthin (Nx) (Fig. 4b). In these organisms, Nx is the precursor of the major light-harvesting carotenoids (ie fucoxanthin, peridinin and vaucheriaxanthin) and, in diatoms like *P. tricornutum*, also of Ddx, which is involved in the Ddx/Dtx cycle. Single nucleotide variants of the *vdl1* gene were overexpressed in two different *P. tricornutum* ecotypes, leading to an increase in the downstream product Ddx and directing the pigments towards fucoxanthin biosynthesis (Li et al. 2024) (Fig. 4b). Also, the transient expression of the *P. tricornutum vdl1* genes in tobacco (*Nicotiana benthamiana*) leaves was observed to induce the accumulation of trans-Nx, confirming the catalytic activity (Dautermann et al. 2020). These results suggest that the biosynthesis of Nx from Vx evolved independently in Chromalveolates, where VDL generates trans-Nx, while in Viridiplantae neoxanthin synthase (NSY) produces 9′-cis-Nx (Bouvier et al. 2000; Dautermann et al. 2020; Giossi et al. 2020).

VDL2 was also proposed to be involved in carotenoid biosynthesis, being responsible for the conversion of Ddx to allenoxanthin, another specific intermediate product of fucoxanthin biosynthesis, downstream Nx (Bai et al. 2022) (Fig. 4b).

Sequence alignment has been used here to check the conservation of key residues in VDE, VDL and VDR. All VDL and VDR forms maintain the same three domains as VDE, overall suggesting similar structural conservation. Structural modeling suggests that VDL and VDR both maintain a structural organization typical of lipocalins. Among the two key residues involved in the VDE catalytic mechanism identified above, Y198 is conserved in all VDR and VDL sequences (Fig. 4c, d), while D177 is not. The aspartate residue is in fact replaced with glutamate in VDL and VDR, thus maintaining the charge but changing the size (Fig. 4c, d). This conservation of polar/charged residues in the hydrophobic protein cavity is consistent with the hypothesis that VDL/VDR have enzymatic activity similar to VDE. In the case of VDL, the residue change is consistent with experimental data showing similar catalytic activity in carotenoid de-epoxidation (Dautermann et al. 2020; Li et al. 2024), while the difference in residue size could enable accommodation of the different substrates.

The N-terminal domain of VDL and VDR remains Cys-rich and all 10 residues are highly conserved (Fig. 4d). The C-terminal Glu-rich domain is significantly less conserved or even absent in VDL proteins (Fig. 4d). This suggests that VDLs possibly differ from VDE in their pH dependence. Since VDLs are involved in carotenoid biosynthesis and thus must be membrane associated to react with hydrophobic substrates, this observation could indicate that the C-terminal domain in VDE is needed to avoid membrane association under neutral pH, rather than to promote it under acidic conditions.

## VDE activity is modulated by multiple mechanisms

Given the central role of VDE and the xanthophyll cycle in photoprotection, energy dissipation, and pigment biosynthesis, the regulation of this enzyme is critical for maintaining photosynthetic balance and productivity. Consistent with this relevance, the activity of VDE is modulated through multiple interconnected mechanisms that are likely instrumental in ensuring its optimal functioning under varying environmental conditions and impacting photosynthetic productivity.

### Regulation of *vde* gene expression

**Figure 5:**
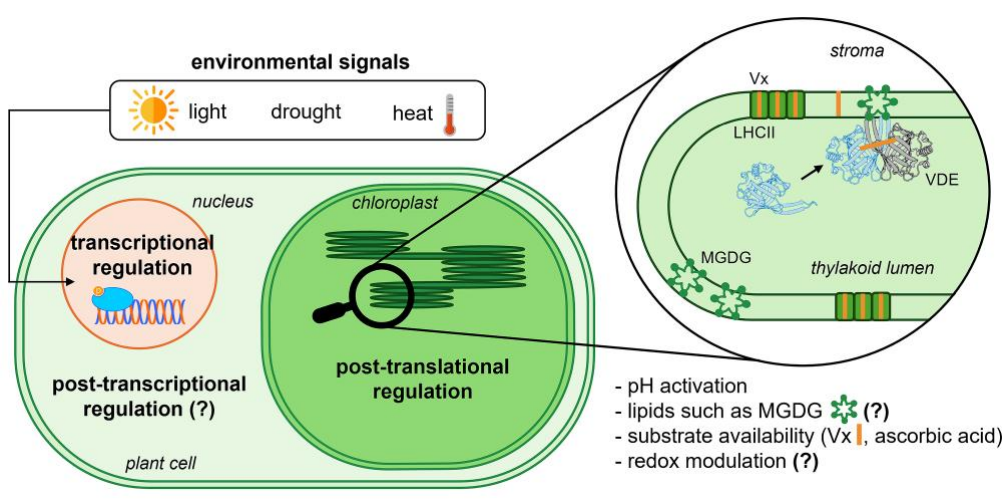
Different levels of VDE regulation. Environmental stimuli can act both on the gene expression and protein synthesis, while physiological changes can induce post-translational modifications *via* metabolic interactions. MGDG: monogalactosyldiacylglycerols.

A first level of VDE regulation is the transcriptional control of the nuclear gene encoding the protein (Fig. 5). Drought stress has been identified as a significant environmental cue inducing the expression of the *vde* gene in different plant species, such as *Cucumis sativus* (cucumber) (Li et al. 2013), *Lycium chinense* (Guan et al. 2015), and *Phyllostachys edulis* (bamboo) (Lou et al. 2022). This can be explained by considering that under water-limiting conditions, plants experience increased excitation pressure on the photosystems due to stomatal closure and reduced CO_2_ assimilation, which elevates the need for photoprotection.

The *vde* gene was shown to be upregulated in different plant species exposed to both low and high temperatures, such as in *C. sativus* (Li et al. 2013), *Triticum aestivum* (Frenette Charron et al. 2005; Shricharan and Kumar 2025) and *P. edulis* (Lou et al. 2022) (Fig. 5). In both cases, this can be explained by considering that temperature stress, whether low or high, can lead to reduced photosynthetic efficiency, excess irradiation, and ROS production (Li et al. 2013), which are counterbalanced by the protective activity of VDE (Lou et al. 2022).

Expectedly, light intensity has been identified as the most impactful environmental factor affecting *vde* transcriptional regulation (Fig. 5). This is consistently observed in experiments across different plant species, such as *P. edulis* (Lou et al. 2022), *C. sativus* (Li et al. 2013), *A. thaliana*, (Jiao et al. 2005), and *Nicotiana tabacum* (tobacco) (Bugos et al. 1999). The expression of the *vde* gene of *A. thaliana VDE* from the same tissue also showed significant diurnal variations in transcript abundance, confirming the impact of the light signal (North et al. 2005). Interestingly, these experiments highlighted that the timing of the expression peaks did not correlate with maximal VDE activity. Similarly, in tobacco leaves, the *vde* transcript levels decreased substantially during leaf expansion, while the amount of the enzyme continued to increase, suggesting that the VDE protein likely has a low turnover (Bugos et al. 1999).

Additional post-transcriptional regulatory mechanisms may play a role in modulating the VDE protein abundance such as mRNA stability, alternative splicing and microRNA-mediated regulation, all of which can contribute to the temporal fine-tuning of protein synthesis under dynamic environmental conditions (Barciszewska-Pacak et al. 2015). While direct evidence for these post-transcriptional mechanisms in the regulation of VDE is currently lacking, these could explain the discrepancies observed between the mRNA levels and protein accumulation.

### Post-translational regulation

Unlike transcriptional control, which may operate on longer timescales, post-translational mechanisms are faster and enable the swift regulation of protein activity through reversible biochemical modifications (Fig. 5). Since environmental conditions are highly dynamic, photosynthetic organisms require constant modulation of their photosynthetic efficiency. On the other hand, the complexes of the photosynthetic apparatus are highly abundant and require a significant investment of energy and nutrients for the synthesis of proteins and pigments. Therefore, it is essential to have mechanisms in place to quickly modulate photosynthesis without involving continuous protein production or degradation, but rather by regulating the activity of the existing complexes.

Consistent with this idea, post-translational regulation plays a critical role in the rapid modulation of VDE activity, enabling photosynthesis to respond swiftly to a dynamic environment, avoiding photodamage upon high light and maximizing light harvesting efficiency when irradiation is limiting (Jahns et al. 2009). As discussed above, a key feature of the VDE regulation is its dependence on the pH of the thylakoid lumen (Fig. 5). In fact, under high light or in any condition where the photosynthetic electron transport activity exceeds the metabolic capacity, the rate of ADP and P_i_ regeneration becomes limiting for ATP synthase activity because of a substrate limitation (Takizawa et al. 2008). The consequent proton accumulation and decrease of lumenal pH is a signal for the activation of the VDE protein, as well as for other photoprotective mechanisms such as NPQ. As discussed above, the thylakoid lumen acidification induces a VDE conformational change (Arnoux et al. 2009; Fufezan et al. 2012). This not only changes protein activity but also controls protein localization. As shown in Fig. 5, VDE at neutral pH is soluble, while at low pH, following a conformational change, it associates with the lumenal side of the thylakoid membrane, a process that is induced at pH values below 6.5 in *A. thaliana* (Bratt et al. 1995; Tian et al. 2019).

This interaction with the membrane is critical for VDE activity, as can be expected considering that its substrate Vx is hydrophobic. VDE activity, however, has been shown to require the presence of specific lipids, in particular monogalactosyldiacylglycerols (MGDG), the most abundant lipid class in thylakoid membranes (Fig. 5). Experiments with liposomes in fact show that VDE is active only when MGDG is present either alone or mixed with other lipids (Latowski et al. 2002; Goss et al. 2005), while being inhibited by the presence of sulfoquinovosyldiacylglycerol (SQDG), an important thylakoid lipid of Chromalveolates (Goss et al. 2009). A specific interaction between VDE and MGDG was indeed inferred (Yamamoto and Higashi 1978; Rockholm and Yamamoto 1996; Jahns et al. 2009), with the latter showing a higher efficiency in the precipitation of VDE compared to other thylakoid membrane lipids (Rockholm and Yamamoto 1996). MGDG is a peculiar lipid that forms inverted hexagonal structures (H_II_), unlike all other thylakoid lipids. Other non-plant lipids with the same structure, such as phosphatidylethanolamine, were also shown to enable VDE activity, demonstrating the need for a specific interaction of the protein with lipids forming these peculiar structures (Latowski et al. 2004). Based on these experiments, the existence of specific MGDG enriched domains in the thylakoid membranes was proposed, representing the docking site for VDE (Jahns et al. 2009). If this is the case, the de-epoxidation rate of Vx could also be limited by its lateral diffusion in those domains (Latowski et al. 2000, 2002). While the essential role of lipids in VDE activity is well established, the potential regulatory impact of modulating its interaction with the membrane, for example by altering the number and size of putative docking sites, remains to be demonstrated.

VDE activity also depends on the availability of its substrates Vx and ascorbic acid (Fig. 5). Vx is primarily bound to light-harvesting complexes (eg LHCII in plants) that release Vx upon lumen acidification (Morosinotto et al. 2002), enabling its conversion into Zx and re-association with the antenna proteins. Carotenoids are essential for the assembly and stability of the LHC structures (Ruban et al. 2002) and depending on the specific binding site they are more or less available for exchange. The Vx molecules bound to sites L2 or V1 can be released from the LHCI in the membrane and thus converted into Zx (Caffarri et al. 2001). Carotenoids bound to the site L1, instead, are essential for protein stability and thus cannot be released without destabilizing the whole protein. Because of this difference in exchangeability, some Vx molecules are excluded from the pool of potential VDE substrates and even prolonged treatments under extremely intense light conditions do not enable a complete conversion into Zx (Perin et al. 2023).

A second consequence of this mechanism is that Zx synthesis could be limited not only by VDE intrinsic activity but also by the kinetics of Vx release from LHC. Indeed, VDE overexpression in plants did not drive faster xanthophyll cycle rates likely because of this limitation in substrate availability (Hieber et al. 2002; Deng et al. 2003). In other organisms, such as the seawater algae *Nannochloropsis oceanica*, VDE overexpression had a direct effect on accelerating the Zx synthesis. This means that Vx was not limiting for conversion, either because there was already a fraction of Vx free in the membrane or because its release from antennas was faster (Michelberger et al. 2025a). This difference with plants is interesting as it shows that during their evolution plants acquired an additional method for the modulation of Zx synthesis.

Experiments performed in *A. thaliana* also showed that the availability of the second substrate, ascorbic acid, can limit VDE activity in vivo, curbing NPQ induction (Müller-Moulé et al. 2002) (Fig. 5). Ascorbic acid availability inside the lumen can be primarily influenced by its membrane permeability and competing metabolic pathways (Bratt et al. 1995). Since ascorbic acid is one of the two major soluble antioxidants in chloroplasts and a cofactor for the antioxidant enzyme-catalyzed reduction of ROS produced by Photosystem I (PSI) in the so-called Mehler-peroxidase reaction (Neubauer and Yamamoto 1994; Asada 1999; Müller-Moulé et al. 2002), oxidative stress can deplete the available ascorbic acid pool through its use in the peroxidase-mediated scavenging of ROS, diverting it away from VDE-dependent reactions (Neubauer and Yamamoto 1994). These competing phenomena are likely to make VDE activity cofactor-limited, at least under some conditions, thereby attenuating the NPQ capacity under suboptimal pH or redox conditions (Neubauer and Yamamoto 1994; Bratt et al. 1995).

Ascorbic acid was also proposed to play a pH-sensitive role in modulating enzyme activity within the thylakoid lumen. Bratt et al. (1995) demonstrated that VDE exhibits a strongly pH-dependent affinity for ascorbic acid, with K_m_ values decreasing significantly as pH drops: from 10 mM at pH 6.0 to 0.3 mM at pH 4.5 (Bratt et al. 1995). This reflects the VDE specificity for the protonated acidic form of ascorbate and suggests that substrate availability in the protonated state also depends on pH. The acidification of the lumen thus not only activates VDE through conformational changes and membrane binding but also increases the proportion of the acid form of ascorbate, thereby amplifying the activity of the enzyme. It is also worth mentioning that, considering concentration and pH, ascorbic acid binding is not necessarily saturated under physiological conditions. This could easily generate cases where only one of the two ascorbic acid binding sites within the VDE dimer is occupied. If this is the case, it would explain the generation of a significant amount of Ax by VDE activity in vivo.

The VDE activity has also been extensively shown to be sensitive to the redox state and reducing agents are widely used to inhibit its activity in vivo (Fig. 5). As discussed above, the VDE protein contains several conserved Cys residues (Hieber et al. 2002; Hallin et al. 2016a) predicted to be involved in disulfide bonds and shown to have a strong impact on protein activity (Simionato et al. 2015) (Fig. 2c, d). Based on this redox-sensitive behavior it has been hypothesized that, in the thylakoid lumen in the dark, the cysteines in VDE could be in the reduced form and upon illumination, following oxygen production from photosynthesis, which makes the lumen more oxidizing, they might promote disulfide bond formation and consequent enzyme activation (Hallin et al. 2015). The Lumen Thiol Oxidoreductase1 (LTO1) was proposed as a candidate for disulfide bond formation in lumenal target proteins: LTO1 has a broad range of substrates, including VDE, suggesting a possible general role for this enzyme in controlling the redox state of lumenal proteins (Karamoko et al. 2013; Simionato et al. 2015). While this hypothesis is interesting, VDE activity has never been shown to be regulated in vivo by modulation of its oxidation state and thus its effective impact remains an open question.

## Open questions on VDE protein properties

Despite the central role of VDE in the regulation of photosynthesis and its impact on productivity, several questions remain about the properties of the protein remain open (Outstanding Questions Box). As an example, it is not clear whether and to what extent VDE homologs from different organisms or lineages are also functionally diversified, tuning enzyme activity or regulation to distinct ecological niches.

The N- and C-terminal domains of the protein are essential for its activity, but a mechanistic explanation of their roles is still missing. The activity of VDE also requires association with thylakoids, but how the protein interacts with the lipid membrane and how this process is regulated remain hypothetical. In parallel, the physiological relevance of a possible redox-dependent modulation of VDE activity warrants further investigation, as it may represent an additional regulatory layer linking chloroplast redox balance to photoprotective responses.

Ultimately, advancing the mechanistic and regulatory understanding of VDE will provide a more robust knowledge framework for efforts to improve the resilience and biomass productivity of photosynthetic organisms in fluctuating light environments.

## Data Availability

There are no supporting data to this article.
